# Assessment of dengue and COVID-19 antibody rapid diagnostic tests cross-reactivity in Indonesia

**DOI:** 10.1186/s12985-021-01522-2

**Published:** 2021-03-11

**Authors:** Marsha S. Santoso, Sri Masyeni, Sotianingsih Haryanto, Benediktus Yohan, Martin L. Hibberd, R. Tedjo Sasmono

**Affiliations:** 1 Dengue Research Unit, Eijkman Institute for Molecular Biology, Ministry of Research and Technology/National Agency for Research and Innovation of the Republic Indonesia, Jl. Diponegoro 69, Jakarta, 10430 Indonesia; 2 Department of Internal Medicine, Faculty of Medicine and Health Sciences, Universitas Warmadewa, Denpasar, Bali, 80235 Indonesia; 3 Raden Mattaher Hospital, Jambi, 36361 Indonesia; 4 Department of Pathogen Molecular Biology, London School of Hygiene and Tropical Medicine, London, WC1E 7HT UK

**Keywords:** COVID-19, RDT, IgG, IgM, Dengue, Specificity, Cross-reactivity

## Abstract

**Background:**

The coronavirus disease 2019 (COVID-19) pandemic remains ongoing around the world, including in areas where dengue is endemic. Dengue and COVID-19, to some extent, have similar clinical and laboratory features, which can lead to misdiagnosis, delayed treatment and patient’s isolation. The use of rapid diagnostic tests (RDT) is easy and convenient for fast diagnosis, however there may be issues with cross-reactivity with antibodies for other pathogens.

**Methods:**

We assessed the possibility of cross-reactivity between SARS-CoV-2 and dengue antibodies by: (1) testing five brands of COVID-19 IgG / IgM RDTs on 60 RT-PCR-confirmed dengue samples; (2) testing 95 RT-PCR-confirmed COVID-19 samples on dengue RDT; and (3) testing samples positive for COVID-19 IgG and/or IgM on dengue RDT.

**Results:**

We observed a high specificity across all five brands of COVID-19 RDTs, ranging from 98.3 to 100%. Out of the confirmed COVID-19 samples, one patient tested positive for dengue IgM only, another tested positive for dengue IgG only. One patient tested positive for dengue IgG, IgM, and NS1, suggesting a co-infection. In COVID-19 IgG and/or IgM samples, 6.3% of COVID-19 IgG-positive samples also tested positive for dengue IgG, while 21.1% of COVID-19 IgM-positive samples also tested positive for dengue IgG.

**Conclusion:**

Despite the high specificity of the COVID-19 RDT, we observed cross-reactions and false-positive results between dengue and COVID-19. Dengue and COVID-19 co-infection was also found. Health practitioners in dengue endemic areas should be careful when using antibody RDT for the diagnosis of dengue during the COVID-19 pandemic to avoid misdiagnosis.

## Background

Severe acute respiratory coronavirus 2 (SARS-CoV-2) emerged in Wuhan, China, causing a respiratory disease, coronavirus disease 2019 (COVID-19), and has now resulted in a global pandemic [1]. The pandemic remains ongoing in many countries, including areas where dengue is endemic, such as Indonesia, which adds a burden to health systems [2, 3]. There were over 130 000 reported cases of dengue in Indonesia in 2019 with an incidence rate of 51.48 cases per 100 000 population, an increase from the previous year’s incidence of 24.75 cases per 100 000 population. As of 21 June, there are 68 000 cases of dengue reported across Indonesia in 2020, while COVID-19 cases continue to increase [4]. As of 26 Jan 2021, there are over one million confirmed cases of COVID-19 in Indonesia [5]. Dengue fever and COVID-19 have similar clinical and laboratory features, which can lead to misdiagnosis, delayed treatment, and isolation [3]. In both cases, patients often report acute fever, myalgia, fatigue, and other flu-like symptoms, as well as present with thrombocytopenia and leukopenia [1, 3]. Most commercial rapid diagnostic tests (RDT) available in the market are for the detection of SARS-CoV-2 antibodies, with relatively high sensitivity and specificity, especially when samples are taken later in the disease progression [6]. However, it is hampered by the apparent cross-reactivity resulting in false-positive results [7]. For dengue, immunochromatographic tests for the detection of dengue virus nonstructural protein 1 (NS1) antigen, IgM, IgG, and IgA antibodies have been developed by a number of commercial companies and have found wide application because of their ease of use and rapidity of results [8, 9].

## Methods

In this study, we assessed the possibility of dengue and SARS-CoV-2 antibody cross-reactivity using three strategies. Firstly, we evaluate the specificity of five COVID-19 RDT brands against 60 well-characterized RT-PCR-confirmed dengue patient’s serum panel. Secondly, we test 95 RT-PCR-confirmed clinical COVID-19 samples on dengue RDT. And thirdly, we test 49 sera from healthy, asymptomatic individuals that are positives for COVID-19 IgG and/or IgM antibodies on dengue RDT (Table 1). The use of archived dengue patients’ samples has been approved by Eijkman Institute Research Ethics Committee, approval number 151/2020, while the use of COVID-19 RT-PCR confirmed samples has been approved by Bali Mandara District Hospital Health Research Ethics Committee, approval number 007/EA/KEPK.RSBM.DISKES/2020, while the use of COVID-19 IgG and/or IgM positive samples has been approved by Raden Mattaher Hospital Research Ethics Committee, approval number S.32/SPE/VII/2020.

**Table 1:** Characteristics of samples used in the study

Categories	Dengue-confirmed samples (N = 60)	COVID-19-confirmed samples (N = 95)*	Healthy/asymptomatic samples (N = 49)
Fever day onset, mean (SD)	5 (1.5)	N/A	N/A
Age, median (IQR)	14 (8–22)	34 (25–46)	43 (34–52)
*Age*	N (%)		
Children ≤ 18 years	35 (58)	2 (2.1)	0 (0.0)
Adults > 18 years	25 (42)	78 (82.1)	49 (100)
*Gender*			
Male	28 (47)	49 (51.6)	34 (69.4)
Female	32 (53)	31 (32.6)	15 (30.6)
*Serotype*			
DENV-1	15 (25)	N/A	N/A
DENV-2	15 (25)	N/A	N/A
DENV-3	20 (33)	N/A	N/A
DENV-4	10 (17)	N/A	N/A
*Immunologic status*			
Primary dengue infection	10 (17)	N/A	N/A
Secondary dengue infection	50 (83)	N/A	N/A
*Presence of anti-dengue antibodies*			
IgG ( +), IgM ( +)	21 (35)	N/A	N/A
IgG ( +), IgM (−)	16 (27)	N/A	N/A
IgG (−), IgM ( +)	14 (23)	N/A	N/A
IgG (−), IgM (−)	9 (15)	N/A	N/A

*Incomplete data for 15 samples

The specificity evaluation of COVID-19 IgG / IgM RDTs against confirmed dengue samples was performed in a laboratory that is internationally certified for Good Clinical Laboratory Practice (GCLP) at the Eijkman Institute, Jakarta, Indonesia. The laboratory testing on RT-PCR-confirmed COVID-19 samples was performed at Bali Mandara and Sanjiwani hospitals, Bali, Indonesia while the laboratory testing on healthy/asymptomatic samples positive for COVID-19 IgG and/or IgM was performed at Siloam Hospital, Jambi, Indonesia, both with appropriate biosafety protocols. Dengue virus (DENV) RNA in the sera were extracted using QIAamp Viral RNA mini kit (Qiagen, Germany) according to the manufacturer’s instructions. Detection and serotyping of DENV was performed using CDC DENV-1-4 real-time RT-PCR assay according to given instructions (Package Insert, KK0128 available at www.cdc.gov/dengue). Molecular detection of SARS-CoV-2 was performed using A*Star Fortitude COVID-19 Real-Time RT-PCR Test and Liferiver Novel Coronavirus (2019-nCoV) Real Time Multiplex RT-PCR (BioVendor, Czech Republic). Immunologic status for dengue was determined using NovaLisa Dengue Virus IgG ELISA (NovaTec Immundiagnostica, Germany), performed according to manufacturer’s instruction. The confirmed-dengue samples were collected from various regions across Indonesia between October 2018 and March 2019, before the initial outbreak of COVID-19. The confirmed-COVID-19 patients were recruited from Bali, Indonesia in May 2020. The healthy/asymptomatic individuals that were positive for COVID-19 IgG and/or IgM were recruited from Jambi, Indonesia from May to July 2020.

The COVID-19 IgG and IgM RDTs that we evaluated were Cellex Inc. (USA), Dynamiker Biotechnology (Tianjin, China), Genbody Inc. (Korea), Standard Diagnostics (Korea), and VivaDiag (Vivacheck Biotech Hangzhou,, China). Each dengue-confirmed sample was tested using different brands of IgG/IgM COVID-19 RDT simultaneously, under uniform conditions, according to their respective manufacturers’ instructions. Positive COVID-19 RDT results were confirmed using SARS-CoV-2 Spike S1-RBD IgG & IgM ELISA Detection Kit (Genscript, USA). The dengue RDT kit used on confirmed COVID-19 samples and healthy/asymptomatic samples was Standard Q Dengue Duo (SD Biosensor, Korea, for the detection of dengue NS1 antigen and IgG and IgM antibodies), while the COVID-19 kit used on the healthy/asymptomatic samples are VivaDiag COVID-19 IgG/IgM.

For COVID-19 RDT specificity evaluation, which is the proportion of non-COVID-19 samples who are correctly identified as negative for SARS-CoV-2 antibodies using the COVID-19 RDTs, sample size was calculated based on the three-step method [10] with an expected specificity of 96%, a precision of 7.5%, and 90% power, which yields 60 presumed COVID-19-negative samples. Overall specificity of each RDT brand, as well as when stratified into variable groups, were compared using Z-tests for proportions. Specificity, confidence intervals, and other statistical analyses were performed using R Studio software, with *p*-value of less than 0.05 denoting statistical significance.

## Results

We observed a high specificity across all COVID-19 RDT brands against dengue-confirmed samples, ranging from 98.3 to 100% and no statistically significant difference in overall performance between brands, or when grouped by age, gender, day of fever onset, DENV serotype, and immunologic status (Table 2). One sample which tested positive for COVID-19 IgG using SD Biosensor Dengue RDT was a 17-year-old male, with fever day onset of 2 days, infected with DENV-4, and tested positive for anti-dengue IgG and IgM. One other sample which tested positive for COVID-19 IgM using Cellex and SD Biosensor Dengue RDT was a 23-year-old male, with fever day onset of 8 days, infected with DENV-2, and tested positive for anti-dengue IgM and negative for anti-dengue IgG. However, neither of these samples tested positive for IgG or IgM using SARS-CoV-2 ELISA, suggesting false-positive results on the RDT.

**Table 2:** Specificity comparison of five brands of COVID-19 RDTs against dengue-confirmed samples

	Specificity (%, 95% CI)	
	IgG	IgM
Cellex	100% (95% CI 92.5–100)	98.3% (95% CI 89.9–99.9)
Dynamiker	100% (95% CI 92.5–100)	100% (95% CI 92.5–100)
Genbody	100% (95% CI 92.5–100)	100% (95% CI 92.5–100)
Standard diagnostics	98.3% (95% CI 89.9–99.9)	98.3% (95% CI 89.9–99.9)
VivaChek	100% (95% CI 92.5–100)	100% (95% CI 92.5–100)
*p*-value	0.404	0.555

Among 95 RT-PCR confirmed COVID-19 patient samples, one patient tested positive for dengue IgM only, another tested positive for dengue IgG only. One patient tested positive for both dengue IgG and IgM as well as NS1, demonstrating the possibility of coinfection of dengue and COVID-19 or recent dengue infection (Table 3).

**Table 3:** Positivity of COVID-19-related samples on dengue NS1, IgG, and IgM RDT

Samples categories	N	Dengue RDT		
		NS1-positive N (%)	IgG-positive N (%)	IgM-positive N (%)
COVID-19 RT-PCR positive	95	1 (1.1)	2 (2.1)	2 (2.1)
COVID-19 IgG RDT positive	33	0 (0.0)	2 (6.1)	0 (0.0)
COVID-19 IgM RDT positive	19	0 (0.0)	4 (21.1)	0 (0.0)

In healthy/asymptomatic samples, out of 33 samples that tested positive for COVID-19 IgG, 2 samples (6.1%) also tested positive for dengue IgG, while out of 19 samples that tested positive for COVID-19 IgM, 4 samples (21.1%) also tested positive for dengue IgG (Table 3).

## Discussion

While there is a possibility of cross-reactivity between SARS-CoV-2 and DENV antibodies, there is good overall performance of COVID-19 RDTs. Another study shows no cross reactivity with other respiratory pathogens that may have similar clinical symptoms as COVID-19 [11]. Apparent cross-reactivity between DENV and SARS-CoV-2 antibodies in dengue RDT has been reported in two confirmed COVID-19 cases in Singapore [7]. However, more in-depth studies are needed to fully understand the interaction between these antibodies.

We found that in terms of specificity, different COVID-19 RDT brands perform similarly to each other, comparable to findings in other studies [12]. However, these studies also show that different RDT brands have varying levels of sensitivity [12]. A meta-analysis of COVID-19 serological tests measured the pooled sensitivity of lateral flow immunoassays to be 66.0% (95% CI 49.3–79.3%) [13]. Taking that sensitivity as a reference, the rapid tests in our study have a negative predictive value (NPV) of 92.2% even at populations with 20% prevalence. The ability to rapidly confirm the absence of COVID-19 would be useful in allocating resources for isolation and protecting the patient from nosocomial COVID-19 infections especially in settings where the patient may have other viral infections that require hospitalization, such as dengue. Additionally, the ability to accurately rule out dengue in acutely febrile patients would quickly lead to the investigation and management of other differential diagnoses such COVID-19 that would require timely isolation and contact tracing.

One of the limitations in this study is we did not assess the sensitivity of COVID-19 RDT data. Another limitation is that we could not conduct repeat testing of dengue antibodies in the COVID-19 confirmed samples and therefore cannot rule out the possibility of recent but not concurrent dengue infection. Additionally, conditions on site unfortunately did not make it possible to follow-up with RT-PCR for DENV. However, the positive anti dengue virus NS1 antigen result has been widely accepted as “confirmed dengue” (WHO SEARO Dengue Guidelines 2011). We also could not assess for cross-reactivity with other coronavirus infections, auto-immune disease, or immunodeficiency. Nevertheless, our data provide important information on the prospective use of these RDTs in regions that are endemic for dengue during the COVID-19 pandemic.

## Conclusion

Our study observed that despite the high specificity of the COVID-19 RDT, cross-reactions and false-positive results between dengue and COVID-19 are very likely. Dengue and COVID-19 co-infection can also occur. To the authors’ knowledge, this is the first study reporting on the performance of COVID-19 rapid tests on dengue-confirmed samples. Our study adds important information on the possible cross-reaction between dengue and COVID-19 in Indonesia, though more studies are needed to further evaluate these interactions. Health practitioners should be careful when using antibody RDT for the diagnosis of dengue during the COVID-19 pandemic to avoid misdiagnosis.

## Data Availability

RDT evaluation raw data available upon request.

## Abbreviations

COVID-19: coronavirus disease 2019
DENV: dengue virus
ELISA: enzyme-linked immunosorbent assay
IgA: immunoglobulin A
IgG: immunoglobulin G
IgM: immunoglobulin M
NS1: nonstructural protein 1
RDT: rapid diagnostic test
RNA: ribonucleic acid
RT-PCR: reverse transcription polymerase chain reaction
SARS-CoV-2: severe acute respiratory coronavirus 2
